# Trends of laboratory nonhuman primate licensing in China between 2020 and 2024: A national database analysis

**DOI:** 10.1371/journal.pone.0348130

**Published:** 2026-05-12

**Authors:** Baoxu Ma, Manying Yuan, Wenxian Xiao, Longbao Lv

**Affiliations:** 1 National Resource Center for Non-Human Primates, Kunming Institute of Zoology, Chinese Academy of Sciences, Kunming, Yunnan, China; 2 Kunming College of Life Science, University of Chinese Academy of Sciences, Kunming, Yunnan, China; Zydus Research Center, INDIA

## Abstract

Nonhuman primates are critical translational models in biomedical research due to their genetic, anatomical, and physiological similarities to humans. China, once a dominant global supplier of captive-bred laboratory primates, has long maintained a national licensure system to govern their production and use. Despite this, the overall scope, spatial distribution, and institutional landscape of licensing activities for laboratory primates in China remain insufficiently characterized, limiting data-driven governance and resource planning. This study conducted a comprehensive retrospective analysis of all laboratory primate licenses issued in China between 2020 and 2024, based on data retrieved from the China Laboratory Animal Licensing Management System (LALMS). In total, 430 licenses were analyzed across production and utilization categories, temporal dynamics, geographical distribution, and institutional ownership. Utilization licenses constituted 85% of the total, while production licenses accounted for 15%. Licensing activity expanded progressively until 2023 before declining sharply in 2024. Among 31 provincial-level administrative divisions in mainland China, 28 were authorized to produce or utilize laboratory primates. Beijing and Jiangsu emerged as primary centers of licensing activity, particularly for utilization. In contrast, production licenses were predominantly concentrated in Guangxi and Yunnan. Utilization licenses exhibited broader geographic distribution than production licenses, although licensing activity was heavily concentrated in East China (31%), North China (22%), and South China (19%). The majority of licenses were held by commercial enterprises (60%), followed by universities (22%) and research institutes (18%). These findings define the temporal and spatial landscape of laboratory primate licensing in China and generate critical data to inform resource allocation, improve regulatory transparency, and promote the responsible and sustainable integration of primate models into biomedical research.

## Introduction

Nonhuman primates (hereafter “primates”) constitute the third most taxonomically diverse group of living mammals after Rodentia and Chiroptera, with 521 recognized species distributed across 91 countries [1]. As the closest extant relatives of humans, primates occupy a central role in biomedical research, enabling mechanistic investigation of complex biological systems and serving as vital translational models bridging fundamental discovery and clinical application [2]. Extensive homology in genetic architecture, anatomical organization, physiological processes, and behavioral traits underpins their unique suitability for modeling human health and disease across a wide range of scientific domains [3]. Captive breeding of primates has long supported experimental research on infectious disease pathogenesis, neurodevelopment, cognitive processing, and functional genomics [4]. Primates are widely employed in preclinical evaluation of pharmaceuticals, biologics, vaccines, and medical devices before progression to human trials [5,6]. The COVID-19 pandemic further underscored their critical role in countermeasure development, facilitating studies of viral pathogenesis, host immune responses, and vaccine efficacy in controlled experimental settings [7–11]. The lack of a structurally or functionally equivalent prefrontal cortex in rodent models highlights the unique advantages of primates for investigating neural circuits underlying cognition, executive control, and neuropsychiatric disorders [12,13]. In parallel, experimental studies of complex traits in primates—such as perceptual integration, abstract reasoning, and social interaction—provide critical insights into the evolutionary emergence of human-specific cognitive capacities [14,15].

A broad range of primate species has been established for biomedical research, encompassing genera such as *Macaca*, *Pan (Homo)*, *Papio*, *Aotus*, *Callithrix*, *Saimiri*, *Chlorocebus*, *Saguinus*, and *Cercocebus* [16,17]. While various species offer lineage-specific advantages for targeted applications, rhesus macaques (*Macaca mulatta*) and cynomolgus macaques (*Macaca fascicularis*), both members of the Old World primates, have remained the predominant models for experimental and traditional medical research throughout the past century [18–20]. Their widespread use is attributed to high physiological and immunological relevance, broad experimental compatibility, and logistical feasibility for long-term colony maintenance and reproduction [21–23]. These two species also dominate the global commercial trade in research primates [24]. According to the Convention on International Trade in Endangered Species of Wild Fauna and Flora (CITES) trade database, rhesus and cynomolgus macaques accounted for 88.9% of all legally exported live primates for scientific purposes between 2015 and 2021 [25]. China, currently the second-largest economy and home to approximately 1.41 billion people [26], ranks second in Asia for primate diversity [27]. At present, 29 species are recognized as native to China [28], representing 5.6% (29/521) of extant primate species globally. Despite this diversity, laboratory research in China remains concentrated on rhesus and cynomolgus macaques [29–31]. Rhesus macaques are native to China, occurring widely in central and southern regions, while cynomolgus macaques are non-native, primarily imported from Southeast Asia [29,32]. By the end of 2024, the captive breeding population of laboratory macaques in China was estimated at 200 000–230 000 individuals, with cynomolgus macaques accounting for approximately 90% and rhesus macaques for 10%, according to the China Laboratory Primate Breeding and Development Association. Compared to earlier estimates [30], this reflects a 21%–31% decline in captive-bred macaque populations over the past 12 years (2013–2024).

Following India’s termination of laboratory primate exports in 1978, China emerged as one of the largest suppliers of captive-bred macaques for biomedical and pharmaceutical research [33,34]. Based on CITES trade records, China was the leading exporter of live primates between 2015 and 2021, accounting for 31.3% of all global exports (n = 105 585) [25]. Prior to the COVID-19 pandemic, China served as a major supplier of laboratory macaques to the United States [35], contributing approximately 50% of annual imports for biomedical research [36]. Between 2010 and 2018, China exported an average of 20 147 cynomolgus macaques per year, totaling 181 328 individuals over that nine-year period [37]. In January 2020, in response to emerging concerns regarding the zoonotic origins of SARS-CoV-2, a nationwide prohibition on wildlife trade was enacted in China [29,38,39]. This policy significantly disrupted international trade patterns for laboratory primates, particularly cynomolgus macaques [37,40]. At present, comprehensive and updated publicly available datasets regarding the legal breeding, allocation, and use of laboratory primates in China remain sparse.

As biologically and behaviorally complex species, primates require specialized, species-appropriate care, environmental enrichment, and housing conditions that meet welfare standards in research settings [17]. Unlike conventional laboratory animals that have undergone extensive domestication, laboratory primates—despite multiple generations in captivity—retain behavioral traits and physiological responses characteristic of wild populations [34]. These attributes introduce significant ethical, logistical, and regulatory challenges, necessitating strict oversight of both breeding and research use across many countries. In China, laboratory primate management operates under a dual system of wildlife conservation and laboratory animal regulation. All 29 native primate species are listed under Table in S1 Table of the China Key Protected Wild Animals List and fall under the jurisdiction of the China Wildlife Protection Law (1988, revised 2022), which prohibits their unauthorized capture, trade, or use [41–43]. Furthermore, legal breeding and use of primates for scientific purposes are permitted under a parallel regulatory framework administered by the Ministry of Science and Technology (MOST). The Statute on the Administration of Laboratory Animals (1988, revised 2017) applies to all research animals, including primates, and establishes the foundational ethical and procedural standards for experimental use [44,45]. To further enhance institutional oversight, MOST issued the Regulation on the Management of Laboratory Animal Quality Control in 1997, which established a mandatory licensure system to regulate production and use [46,47]. Licenses are centrally registered with MOST, while provincial enforcement is delegated to the Provincial Departments of Science and Technology (PDST) through their subordinate Administration Offices for Laboratory Animals (AOLAs), which conduct annual evaluations through on-site inspections and institutional reporting [47]. In 2001, MOST, in collaboration with other national ministries, released the Temporary Regulation on the Management of Laboratory Animal Licensing System, which detailed the implementation procedures for licensure and further expanded the regulatory infrastructure [48,49].

To date, most existing research has focused on traditional biodiversity conservation of wild primate populations [27,43,50], global patterns in live primate trade [24,51,52], or broad reviews of laboratory animal policies and regulatory frameworks [44,47,49,53]. However, despite the former role of China as the leading global supplier of captive-bred macaques for biomedical and pharmaceutical research, no comprehensive analysis has been conducted on the national licensing system governing laboratory primates. This study explored the licensing database regulating laboratory primate operation for scientific research, quantified the number and categories of licenses issued by local authorities, evaluated spatiotemporal trends in licensing activity from 2020 to 2024, compared certificate distributions across provincial-level divisions, and identified the geographical hotspots for each license category. These findings provide a foundational reference for understanding the current licensing framework and offer data-driven guidance for advancing regulatory oversight and supporting more efficient allocation of laboratory primate resources.

## Materials and methods

### Data acquisition

The Laboratory Animal Licensing Management System (LALMS) database (https://www.lascn.net/Category_1377/Index.aspx) serves as the national repository for laboratory animal license records from 31 provincial-level administrative divisions of mainland China, including provinces, municipalities, and autonomous regions. The database is maintained by the National Laboratory Animal Data Resource Center and Guangdong Provincial Biotechnology Research Institute. LALMS is updated annually to reflect the current status of all laboratory animal licenses; however, public access is restricted to records from the most recent five-year period. Each entry includes license category, serial number, licensed entity, permitted scope, facility address, issuing authority, year of issuance, approved animal taxa, and the effective and expiration dates. In accordance with the 1997 Regulation, licenses are classified into two primary categories—production and utilization—as defined in Table 1.

**Table 1:** Categories of laboratory animal licenses issued in China.

Type	Description
**Production license**	Laboratory animal breeders: “any institutions engaged in breeding and commercial trading of laboratory animals must obtain a production license from MOST”
**Utilization license**	Laboratory animal users: “any institutions involved in animal experiments and using laboratory animals for assessment of pharmaceutical and biological products must acquire a utilization license from MOST”

### Data analysis

A structured analytical workflow was implemented to examine valid laboratory primate licenses in China, including extraction of records from the LALMS database, standardized data compilation, temporal filtering, and keyword-based screening. Relevant records were extracted from the LALMS database and compiled into a standardized spreadsheet for data processing. As of 31 December 2024, the database contained 3 404 laboratory animal licenses. Licenses issued within the five-year period from 1 January 2020–31 December 2024 were selected, yielding 2 878 valid records. A secondary screening was then conducted to exclude licenses unrelated to primates. Filtering was conducted using keywords such as “monkey”, “laboratory monkey”, “nonhuman primate”, “primate”, “rhesus macaque”, “cynomolgus macaque”, and “common marmoset”. According to regulations governing laboratory animal licensing in China [48], production and utilization licenses remain valid for five years and may be renewed upon expiration. Licenses included in the analysis corresponded to distinct independent entities, and no entity was counted more than once during the 2020–2024 study period. This process identified a final dataset of 430 laboratory primate licenses, which were subsequently summarized and analyzed.

A total of 430 licenses were included for summary and analysis. Each license was reviewed for key variables, including license type, licensed entity, facility address, issuing provincial-level authority, year of issuance, approved animal taxa, and effective and expiration dates. Statistical analysis and data visualizations were performed using GraphPad Prism (GraphPad Software, San Diego, CA, USA; https://www.graphpad.com/) and R v3.6.3 (R Foundation for Statistical Computing, Vienna, Austria; https://www.r-project.org/). A chi-square goodness-of-fit test was applied to assess the distribution of license issuance by year and geographic region. Statistical significance was defined as *p* < 0.05.

## Results

### Overall landscape

**Fig 1:**
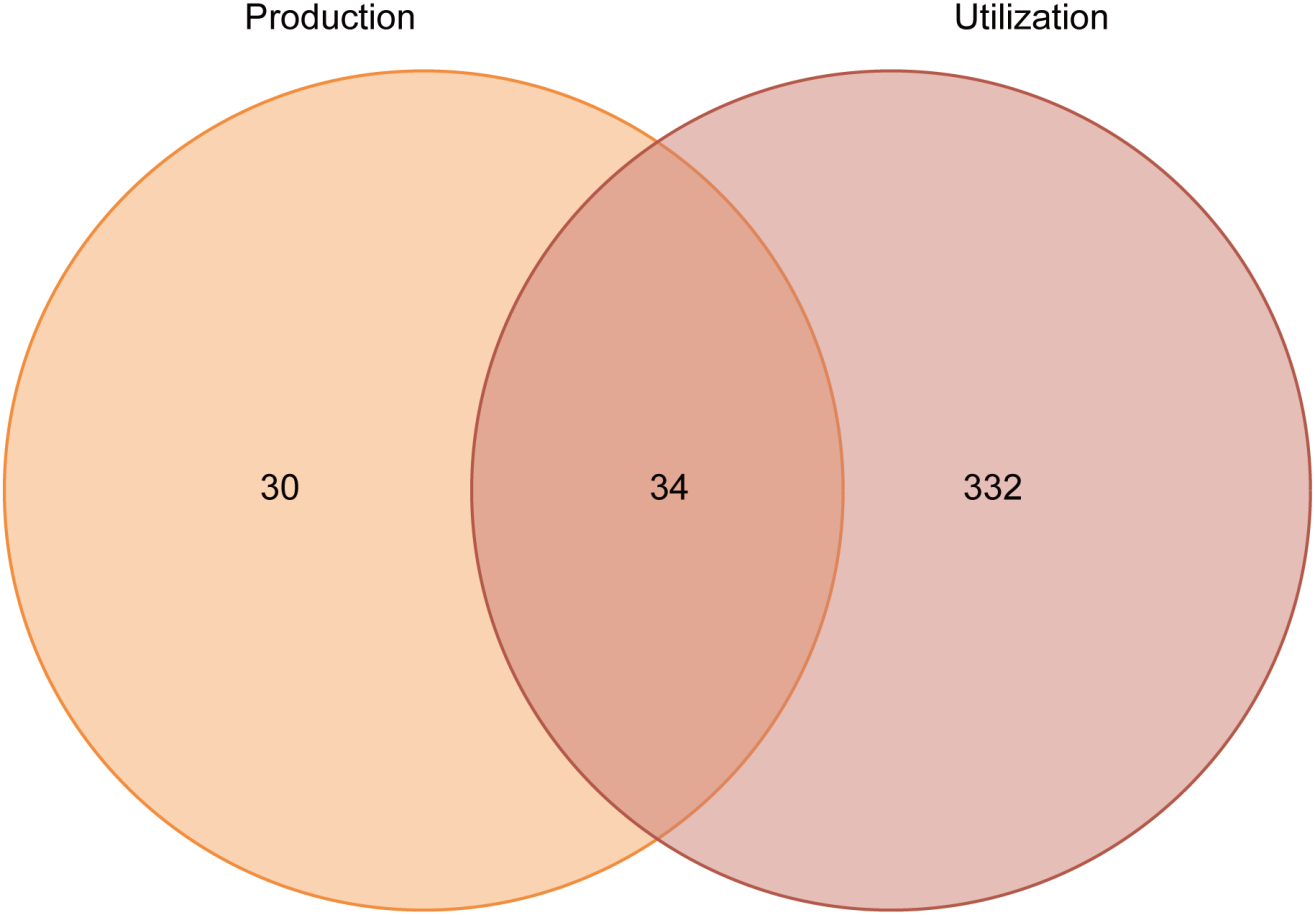
Overview of laboratory primate licenses issued by Provincial Departments of Science and Technology (PDSTs) as of the end of 2024. Left circle (light orange) represents production license; right circle (light pink) represents utilization license; overlapping area represents entities authorized for both production and utilization of laboratory primates.

Between 2020 and 2024, a total of 430 laboratory primate licenses were issued by the PDSTs, representing approximately 15% of all laboratory animal licenses issued during the same period. In terms of license category, utilization licenses constituted the majority, with 366 licenses (85%) issued for experimental use of primates. In contrast, only 64 production licenses (15%) were granted for laboratory primate breeding and commercial purposes. Dual-category licensing that encompassed both production and utilization was uncommon, with only 34 licenses (8%) granted to entities authorized to conduct both activities (Fig 1). Nevertheless, these dual-licensed entities accounted for more than half of all production licenses (34/64; 53%), indicating that many production facilities also held authorization for utilization. In contrast, entities holding both license types represented only 9% of those authorized for utilization (34/366).

**Fig 2:**
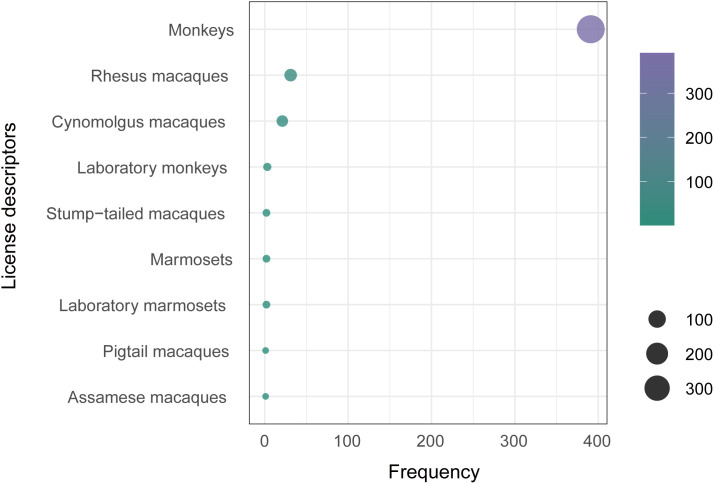
Descriptors listed in laboratory primate licenses. Circle size and different colors represent the frequency of occurrence.

Regarding descriptive terms, 9 distinct terms were recorded across the 430 laboratory primate licenses, including “Monkeys”, “Laboratory monkeys”, “Marmosets”, “Laboratory marmosets”, “Rhesus macaques”, “Cynomolgus macaques”, “Assamese macaques”, “Pigtail macaques”, and “Stump-tailed macaques”. Among all taxonomic descriptors listed on the laboratory primate licenses, “Monkeys” appeared most frequently (Fig 2). This terminology aligns with common usage in China, where primates used for scientific purpose are broadly referred to as “laboratory monkeys”. According to the Chinese National Standard GB/T 39759–2021 Laboratory Animal-Terminology, “laboratory monkeys” are defined as primates derived from wild populations through domestication, including species such as *Macaca mulatta* (rhesus macaques), *Macaca fascicularis* (cynomolgus macaques), and *Callithrix jacchus* (common marmosets). Although most licenses do not specify the exact primate taxa authorized for production or use, the widespread use of the general term “monkey” refers to these commonly used species in Chinese biomedical research settings.

### Trends in production and utilization licenses

**Fig 3:**
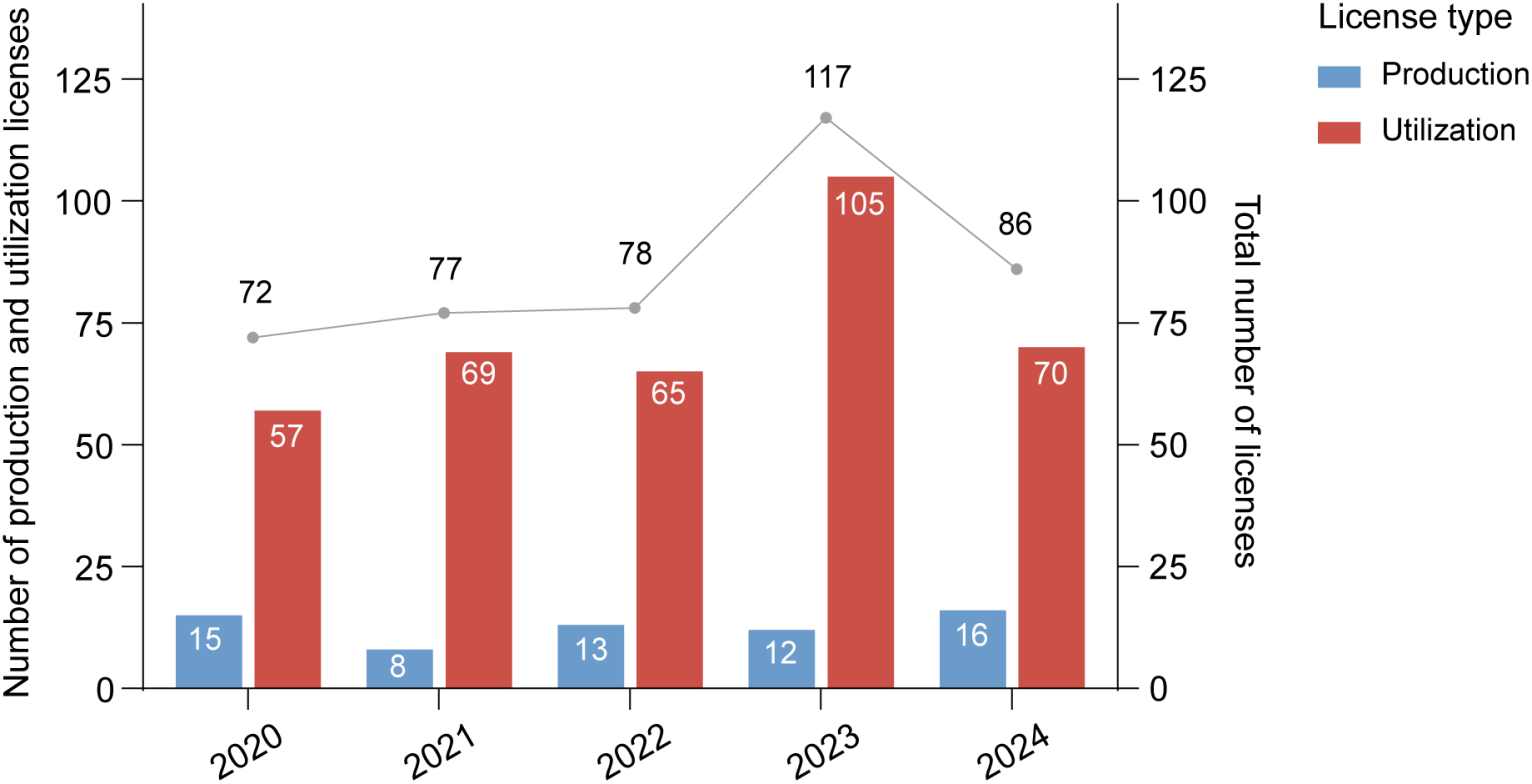
Annual number of laboratory primate production and utilization licenses issued in China from 2020 to 2024. Production licenses (blue) represent entities authorized to breed, maintain, supply, transport, and trade laboratory primates and related products. Utilization licenses (red) represent entities permitted to use laboratory primates and related products for scientific research.

The number of laboratory primate licenses issued in China between 2020 and 2024 exhibited marked year-to-year variation (Fig 3). Overall license distribution was significantly non-uniform over the five-year period (χ² = 15.139, df = 4, *p* < 0.01). From 2020 to 2022, issuance increased gradually, followed by a rapid rise in 2023 that marked the highest annual total of the study period (*n* = 117). However, this surge was followed by a steep decline in 2024, with only 86 licenses issued—a 26% reduction compared to the previous year. Across the five-year period, annual license totals remained below 100 in four out of five years, with 2023 as the only exception. On average, 86 laboratory primate licenses were issued per year.

License trends also differed by category. Notably, utilization licenses far outnumbered production licenses throughout the study period (Fig 3). The annual count of utilization licenses fluctuated, averaging 73.2 ± 18.5 (range = 57–105), peaking in 2023 (*n* = 105), followed by a marked decline in 2024 (*n* = 70). In contrast, production licenses remained relatively stable throughout the study period, with annual counts consistently below 20 (average 12.8 ± 3.11; range = 8–16). The lowest number was recorded in 2021 (*n* = 8), followed by a steady increase, reaching a five-year high in 2024 (*n* = 16).

### Overall distribution of licensing activity

**Fig 4:**
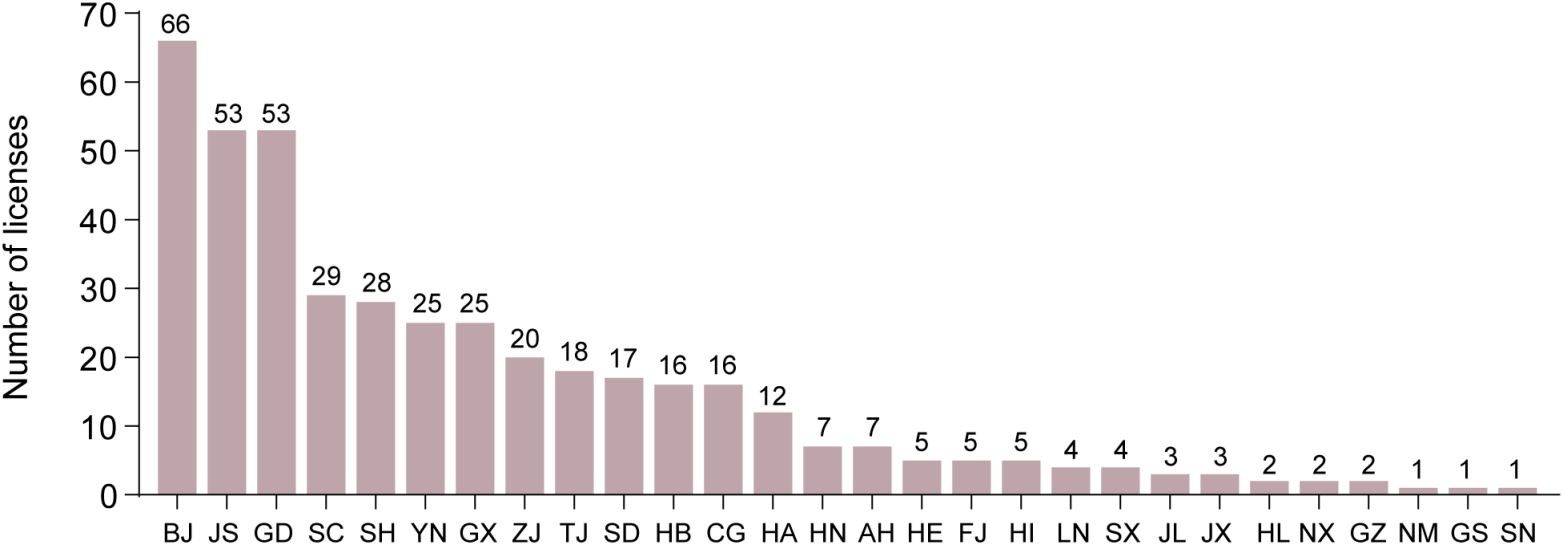
Number of laboratory primate licenses issued across provincial-level administrative divisions in China from 2020 to 2024. Administrative division codes: BJ-Beijing; JS-Jiangsu; GD-Guangdong; SC-Sichuan; SH-Shanghai; YN-Yunnan; GX-Guangxi; ZJ-Zhejiang; TJ-Tianjin; SD-Shandong; HB-Hubei; CQ-Chongqing; HA-Henan; HN-Hunan; AH-Anhui; HE-Hebei; FJ-Fujian; HI-Hainan; LN-Liaoning; SX-Shanxi; JL-Jilin; JX-Jiangxi; HL-Heilongjiang; NX-Ningxia; GZ-Guizhou; NM-Inner Mongolia; GS-Gansu; SN-Shaanxi.

Between 2020 and 2024, laboratory primate licenses were issued across 28 provincial-level administrative divisions in China, including provinces, municipalities, and autonomous regions (Fig 4). The distribution of licenses was highly uneven among these regions (χ² = 530.856, df = 27, *p* < 0.0001). Beijing (BJ) recorded the highest number of licenses issued over the five-year period (*n* = 66), followed by Jiangsu (JS) and Guangdong (GD) (53 licenses each). Other major contributors included Sichuan (SC) (*n* = 29), Shanghai (SH) (*n* = 28), Yunnan (YN) (*n* = 25), Guangxi (GX) (*n* = 25), Zhejiang (ZJ) (*n* = 20), Tianjin (TJ) (*n* = 18), and Shandong (SD) (*n* = 17). Collectively, these top 10 provincial-level administrative regions accounted for 334 licenses, representing 78% of all laboratory primate licenses issued nationwide. In contrast, the bottom 10 regions each issued fewer than five licenses, reflecting a highly concentrated geographic distribution of licensing activity.

### Geographical distribution of production and utilization licenses

Mainland China is divided into seven major geographical regions—Northeast, East, North, Central, South, Southwest, and Northwest China—each encompassing specific provincial-level administrative divisions (Table in S2 Table). The distribution of laboratory primate licenses across these regions exhibited pronounced variation (Table in S3 Table), with total licenses issued ranking as follows: East China (133, 31%)> North China (94, 22%)> South China (83, 19%)> Southwest China (72, 17%)> Central China (35, 8%)> Northeast China (9, 2%)> Northwest China (4, 1%). East and North China together accounted for more than half of all national licensing activity. In contrast, Northwest China contributed only a negligible fraction of licenses, suggesting limited infrastructure and minimal engagement in primate-related research and breeding in that region.

**Fig 5:**
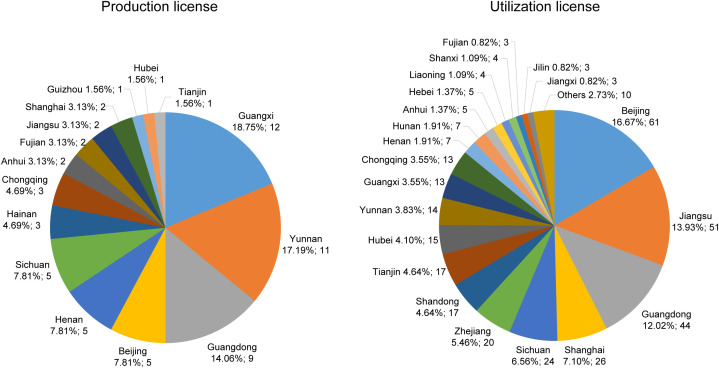
Proportion of laboratory primate licenses by category across provincial-level administrative divisions in China. “Others” includes Hainan, Heilongjiang, Inner Mongolia, Ningxia, Gansu, Guizhou and Shaanxi, each of which issued fewer than three utilization licenses during the study period.

The spatial distribution of laboratory primate licenses also varied substantially across license categories and provincial-level administrative divisions (Fig 5). Between 2020 and 2024, production licenses were issued in only 15 out of 31 divisions. Guangxi (12, 19%), Yunnan (11, 17%), and Guangdong (9, 14%) issued the highest numbers, each accounting for more than 10% of the national total (Fig 5, left panel). Together, these three provincial-level administrative divisions issued approximately 50% of all production licenses between 2020 and 2024. In contrast, utilization licenses were distributed across 28 divisions, with the majority concentrated in Beijing (61, 17%), Jiangsu (51, 14%), and Guangdong (44, 12%), together accounting for nearly half of all such licenses nationwide (Fig 5, right panel). Notably, no licenses were recorded in Qinghai, Xinjiang, or Tibet, indicating the absence of authorized primate-related activities in these regions. Although Guangxi accounted for the highest share of production licenses, it contributed just 4% of all utilization licenses nationwide, highlighting a stark divide between breeding capacity and research activity.

### Identification of licensed entities

Licensed entities in this study were categorized based on their institutional attributes, including profit-oriented enterprises (e.g., contract research organizations, pharmaceutical companies, and private laboratory primate suppliers), government-funded research institutes, and publicly funded universities. The distribution of laboratory primate licenses was highly skewed toward profit-oriented enterprises, which held 258 licenses, accounting for 60% of all licensed entities (Table in S4 Table). Research institutes and universities held 18% and 22% of licenses, respectively.

**Fig 6:**
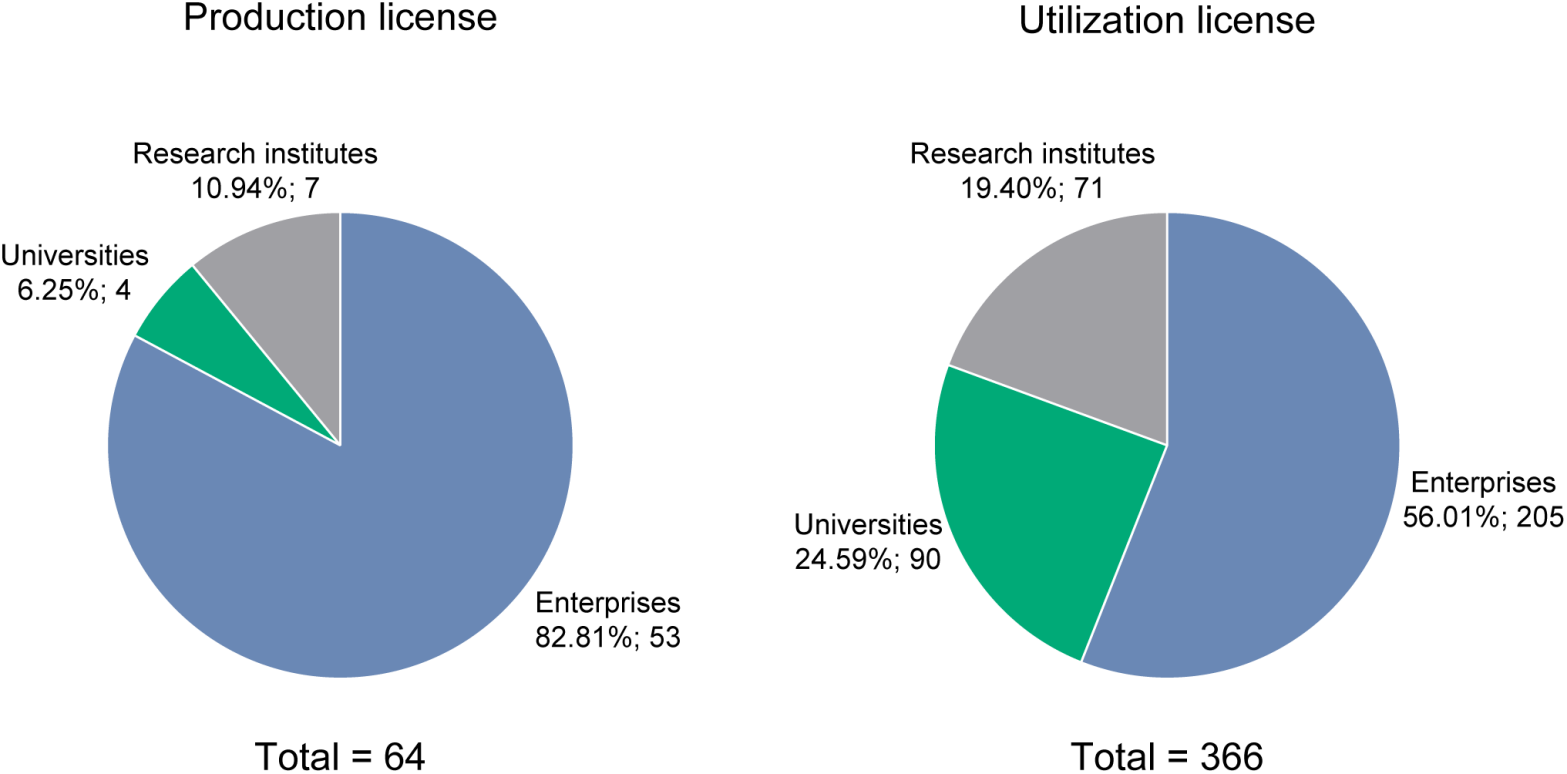
Distribution of production and utilization licenses for laboratory primates by entity type. Enterprises (blue) refer to commercial institutions, including contract research organizations (CROs), pharmaceutical companies, and private laboratory primate suppliers. Research institutes (gray) refer to government-funded institutions focused on specific scientific fields. Universities (green) refer to publicly funded institutions engaged in higher education, academic research, and public service.

The distribution of license types varied markedly across entity categories (Fig 6). Between 2020 and 2024, profit-oriented enterprises held 53 of the 64 production licenses for laboratory primates, representing 83% of the total and substantially exceeding the combined share of research institutes and universities (Fig 6, left panel). Four universities—Yunnan Agricultural University (YAU), Tianjin Medical University (TMU), Kunming University of Science and Technology (KUST), and Tsinghua University (THU)—were authorized to produce laboratory primates and together accounted for 6% of all production licenses. For utilization of laboratory primates, 205 licenses (56%) were issued to commercial enterprises (Fig 6, right panel), while the remaining 44% were issued to publicly funded universities (*n* = 71) and research institutes (*n* = 90), reflecting broader non-profit institutional participation in primate-based scientific research.

## Discussion

As the closest extant relatives to humans, primates serve as pivotal models for investigating complex biological processes and advancing biomedical innovation [54]. Their high translational relevance has been particularly evident during global health emergencies, such as the COVID-19 pandemic, reaffirming the critical role of primates in bridging foundational research and clinical application [55–57]. Consequently, the global scientific community has maintained strong interest in the production and utilization of primates, especially in countries that play leading roles in the international laboratory primate supply chain [30,58,59]. Although China previously functioned as a major supplier of captive-bred monkeys, public access to data on primate-based research and production has remained limited since the 2020 suspension of wildlife exports [29]. This study leveraged a comprehensive, nationwide database to assess licensing activities for laboratory primates in mainland China, providing a systematic evaluation of national licensing trends in the post-pandemic era. This research delineated the regulatory landscape governing laboratory primates in China, offering quantitative insights into market scale, regional licensing disparities, temporal issuance trends, and institutional engagement in the production and utilization sectors. These findings provide a foundational reference for understanding the current licensing framework in China, offering data-driven insights to advance regulatory oversight and optimize the allocation of laboratory primate resources. Moreover, our study fosters transparency in primate management to address public concerns. It also offers distinct guidance for cross-border collaborations and ethical compliance, highlighting the implications of China’s regulatory landscape for international researchers and the global supply chain.

Between 2020 and 2024, 430 laboratory primate licenses were issued in China, representing a substantial increase relative to the 246 licenses issued during the 2014–2018 period [60]. This expansion likely reflects intensified demand associated with the COVID-19 pandemic and the accelerated growth of biotechnology research and development. Non-human primates remain among the most informative animal models for the study of infectious diseases due to their close phylogenetic relationships with humans and high translational relevance [61,62]. The emergence of the COVID-19 pandemic at the end of 2019 initiated a period of intense global effort focused on vaccine development, antiviral therapeutics, and experimental modeling of viral pathogenesis. In parallel, major investments in primate research infrastructure and breeding facilities have been implemented in China since 2019, supported by both central and provincial authorities [58,59,63]. Representative examples include the National Resource Center for Non-Human Primates and the National Research Facility for Phenotypic and Genetic Analysis of Model Animals (Primate Facility) under the Chinese Academy of Sciences (CAS) [64–66]. Establishment of these national facilities has stimulated the expansion of institutional capacity for primate-based research and breeding, thereby contributing to the observed increase in licensing activity.

Despite this growth, laboratory primate licenses continue to represent only a modest proportion of total national laboratory animal licensing. During 2014–2018, laboratory primate licenses accounted for 4% of all licenses issued (246/6 387) [60], whereas the proportion increased to 15% (430/2 878) during 2020–2024. The limited number of laboratory primate licenses can be attributed to both ethical and regulatory constraints. Primates are used far less frequently than rodents or dogs in experimental research due to ethical considerations associated with their cognitive complexity and social behavior [18]. In addition, all primate species are classified under the China Key Protected Wild Animals List and are subject to stringent supervision under the China Wildlife Protection Law. As of 2024, a total of 11 laws and regulations have been enacted to govern the production and use of laboratory primates (Table in S5 Table). These regulatory measures collectively restrict the scale of primate use in scientific research.

At present, most laboratory primates in China are used in preclinical safety assessment, vaccine and therapeutic development, human disease modeling, and translational biomedical studies. Although large-scale primate breeding and experimental use were initiated later than in regions, such as the United States, the United Kingdom, Japan, and the European Union, substantial national commitment has been directed toward the establishment of integrated platforms that combine primate production with research utilization to enhance experimental efficiency and reinforce regulatory oversight. Continued expansion of research infrastructure and improvement of supervisory frameworks are expected to drive further development of standardized breeding systems, more rigorous ethical review procedures, strengthened animal welfare protections, increasingly precise generation of primate disease models, and stronger alignment with international programs in innovative drug discovery and development. These developments are anticipated to provide sustained support for biomedical innovation at both national and global scales.

Analysis of licensing data indicated that 28 of 31 provincial-level administrative divisions in mainland China were authorized for laboratory primate production or utilization, with only Xinjiang, Qinghai, and Tibet lacking such authorization. Licensing activity demonstrated pronounced spatial clustering, with the highest concentrations observed in Beijing and Jiangsu. Regions with concentrated licensing activity have concurrently developed more robust oversight frameworks. Beijing and Jiangsu, which issued 15 and 11 region-specific standards (DBs) respectively, have surpassed other provinces in subnational regulatory development. Beijing not only enacted the first local legislation on laboratory animals but also released the earliest comprehensive guidelines for common marmoset production and use. These advancements suggest that intensified licensing activity is associated with stricter supervision. In contrast, variability in enforcement across provinces appears linked to regional disparities in economic development [47]. The comparatively advanced biomedical sectors of Beijing and Jiangsu support stronger regulatory infrastructure [67–69]. While Beijing leads in regulatory approvals, Guangxi, Yunnan, and Guangdong have emerged as key centers for production licensing. This is likely driven by favorable geographic conditions, environmental suitability, and resource availability on rhesus macaques [70].

Analysis of licensing entities revealed a highly commercialized structure of the laboratory primate sector in China, with profit-oriented enterprises holding more than 80% of production licenses. According to the latest statistical analysis by the China National Resource Center for Non-Human Primates, the 10 largest laboratory monkey holders are all commercial entities. This pattern is consistent with our analysis of entity attributes, highlighting a strongly commercialized and market-driven structure in the Chinese laboratory primate sector. This may reflect the fact that major pharmaceutical companies and contract research organizations have secured deep access to laboratory monkey supply chains through acquisitions and equity-based control. For example, WuXi PharmaTech acquired Guangdong Blooming-Spring Biological Technology for RMB 804 million, securing approximately 20 000 live monkeys for experimental use. Joinn Laboratories completed full acquisitions of Guangxi Weimei and Yunnan Yingmao in 2022, adding another 20 000 monkeys to its holding capacity. Through such transactions, industrial capital has concentrated control over laboratory primate resources, reducing turnover in the open market, exacerbating supply shortages, and driving sustained price increases.

Analysis also identified “Monkeys” as the most frequently used descriptor in license records. Although broad descriptors such as “Monkeys”, “Laboratory monkeys”, or “Laboratory primates” may comply with national registration standards, their widespread use raises multiple concerns. Generalized labeling compromises transparency and hinders effective oversight and monitoring of laboratory primates. It also creates ambiguity for researchers and breeders attempting to determine which specific taxa are authorized for use. This lack of specificity may further undermine public safety measures and conservation efforts for native wild primate populations [71]. Non-governmental organizations have long criticized insufficient transparency in the use of animals for scientific purposes. In response, licensing systems and practices in the European Union and United States have incorporated measures to enhance openness and accountability over the past decade [72,73]. As China strives to align laboratory animal regulation with international standards and develop world-class research infrastructure, it is imperative to improve the granularity of license documentation. Specifically, local authorities should prioritize the inclusion of taxonomically precise terminology in all laboratory primate licenses.

Although this study provides a comprehensive overview of laboratory primate licensing across mainland China from 2020 to 2024, it does not constitute a complete national inventory due to several limitations. First, analysis was restricted to the 31 provincial-level administrative divisions of mainland China, with Hong Kong, Macao, and Taiwan excluded as they operate under separate regulatory frameworks not included in the LALMS database. Second, the dataset only covered the most recent five-year period (2020–2024), limiting the ability to assess longitudinal trends—particularly licensing dynamics before and after the COVID-19 pandemic. Third, most licensed entities do not report species-level permit data, impeding efforts to determine the number of authorizations involving specific taxa such as cynomolgus macaques (*Macaca fascicularis*) or rhesus macaques (*Macaca mulatta*). Despite these constraints, this retrospective study constitutes one of the first published analyses of laboratory primate licensing activity in China in recent years. Licensing remains a critical tool for upholding high welfare standards and promoting ethical oversight of primate use in research. Ongoing surveillance of laboratory primate licensing is essential for ensuring effective regulatory governance. Future laboratory animal licensing policies in China and internationally should respond to public concerns regarding laboratory primate welfare by instituting regular assessments of licensing activity, prioritizing oversight in high-licensing regions, and enhancing the transparency and accessibility of licensing data.

## Conclusions

As the closest extant relatives of humans, laboratory primates play a pivotal role in biomedical research as critical translational models that bridge basic scientific discovery and clinical application. China remains one of the largest global suppliers of captive-bred primates for scientific use, despite the nationwide ban on wildlife trade enacted in 2020, and its laboratory primate sector continues to expand. This study, based on a comprehensive analysis of laboratory primate licensing, identified a highly uneven, geographically clustered, and increasingly commercialized licensing system shaped by national policy and research demand. At the license type level, utilization licensing activity consistently exceeded that of production licensing, indicating a limited number of supply entities for laboratory primates. Spatial analysis revealed heavily concentrated licensing activity in East and North China, particularly in Beijing and Jiangsu, where localized regulatory frameworks have been developed. Furthermore, comparative analysis indicated that commercial entities dominate both production and utilization licensing, raising concerns regarding restricted supply and inflated costs, which may hinder publicly funded research. While a regulatory basis for laboratory primate production and use has been established in China, further investigation offers a critical opportunity to strengthen ethical oversight and contribute to international standards. Enhanced monitoring of laboratory animal licensing—particularly for primates—will be essential to ensure regulatory accountability, protect animal welfare, and enable the responsible advancement of biomedical research.

## Supporting information

S1 Table Native extant primate species in China and their current conservation status. (DOCX)

S2 Table Division of mainland China into seven major geographical regions. (DOCX)

S3 Table Laboratory primate licenses issued across the seven major geographical regions of China. (DOCX)

S4 Table Laboratory primate licenses categorized by primary institutional attributes. (DOCX)

S5 Table Principal laws and regulations relating to the management of laboratory primate in China. (DOCX)
