# Supplementary material for: Trends of laboratory nonhuman primate licensing in China between 2020 and 2024: A national database analysis
Source: PLoS One. 2026 May 12;21(5):e0348130. doi: 10.1371/journal.pone.0348130 (PMC13166922; doi:10.1371/journal.pone.0348130)
Supplement: S1 Table — (DOCX) [file pone.0348130.s001.docx]

**S1 Table. Native extant primate species in China and their current conservation status.**

| **Scientific Name** | **Common Name** | **China Conservation Level** | **IUCN Red**  **List** | **CITES**  **Appendix** |
| --- | --- | --- | --- | --- |
| *Macaca arctoides* | Stump-tailed macaque | II | VU | II |
| *Macaca assamensis* | Assamese macaque | II | NT | II |
| *Macaca cyclopis* | Formosan rock macaque | I | LC | II |
| *Macaca leonina* | Northern pig-tailed macaque | I | VU | II |
| *Macaca leucogenys* | White-cheeked macaque | II | EN | II |
| *Macaca mulatta* | Rhesus macaque | II | LC | II |
| *Macaca munzala* | Arunachal macaque | II | EN | II |
| *Macaca thibetana* | Tibetan macaque | II | NT | II |
| *Semnopithecus schistaceus* | Nepal gray langur | I | LC | I |
| *Trachypithecus crepusculus* | Indochinese gray langur | I | EN | II |
| *Trachypithecus francoisi* | François’s langur | I | EN | II |
| *Trachypithecus phayrei* | Phayre’s leaf-monkey | I | EN | II |
| *Trachypithecus pileatus* | Capped langur | I | VU | I |
| *Trachypithecus leucocephalus* | White-headed black langur | I | CR | II |
| *Trachypithecus shortridgei* | Shortridge’s langur | I | EN | I |
| *Rhinopithecus bieti* | Black snub-nosed monkey | I | EN | I |
| *Rhinopithecus brelichi* | Grey snub-nosed monkey | I | CR | I |
| *Rhinopithecus roxellana* | Golden snub-nosed monkey | I | EN | I |
| *Rhinopithecus strykeri* | Myanmar snub-nosed monkey | I | CR | I |
| *Nycticebus bengalensis* | Bengal slow loris | I | EN | I |
| *Nycticebus pygmaeus* | Southern pygmy loris | I | EN | I |
| *Hoolock hoolock* | Western hoolock gibbon | I | EN | I |
| *Hoolock leuconedys* | Eastern hoolock gibbon | I | VU | I |
| *Hoolock tianxing* | Skywalker hoolock gibbon | I | EN | I |
| *Hylobates lar* | Lar gibbon | I | EN | I |
| *Nomascus concolor* | Black-crested gibbon | I | CR | I |
| *Nomascus nasutus* | Cao-vit gibbon | I | CR | I |
| *Nomascus hainanus* | Hainan gibbon | I | CR | I |
| *Nomascus leucogenys* | Northern white-cheeked gibbon | I | CR | I |
