# Supplementary material for: Trends of laboratory nonhuman primate licensing in China between 2020 and 2024: A national database analysis
Source: PLoS One. 2026 May 12;21(5):e0348130. doi: 10.1371/journal.pone.0348130 (PMC13166922; doi:10.1371/journal.pone.0348130)
Supplement: S2 Table — (DOCX) [file pone.0348130.s002.docx]

**S2 Table. Division of mainland China into seven major geographical regions.**

| **Region** | **Provincial-level administrative divisions** |
| --- | --- |
| Northeast | Heilongjiang, Jilin, Liaoning |
| East | Jiangsu, Shanghai, Zhejiang, Shandong, Anhui, Fujian, Jiangxi |
| North | Beijing, Tianjin, Hebei, Shanxi, Inner Mongolia |
| Central | Hubei, Henan, Hunan |
| South | Guangdong, Guangxi, Hainan |
| Southwest | Sichuan, Guizhou, Yunnan, Chongqing, Tibet |
| Northwest | Shaanxi, Gansu, Qinghai, Ningxia, Xinjiang |
