# Supplementary material for: Trends of laboratory nonhuman primate licensing in China between 2020 and 2024: A national database analysis
Source: PLoS One. 2026 May 12;21(5):e0348130. doi: 10.1371/journal.pone.0348130 (PMC13166922; doi:10.1371/journal.pone.0348130)
Supplement: S3 Table — (DOCX) [file pone.0348130.s003.docx]

**S3 Table. Laboratory primate licenses issued across the seven major geographical regions of China.**

| **Region** | **Number of licenses** | **Percentage of total %** |
| --- | --- | --- |
| East | 133 | 30.93% |
| North | 94 | 21.86% |
| South | 83 | 19.30% |
| Southwest | 72 | 16.74% |
| Central | 35 | 8.14% |
| Northeast | 9 | 2.09% |
| Northwest | 4 | 0.93% |
