# Supplementary material for: Trends of laboratory nonhuman primate licensing in China between 2020 and 2024: A national database analysis
Source: PLoS One. 2026 May 12;21(5):e0348130. doi: 10.1371/journal.pone.0348130 (PMC13166922; doi:10.1371/journal.pone.0348130)
Supplement: S4 Table — (DOCX) [file pone.0348130.s004.docx]

**S4 Table. Laboratory primate licenses categorized by primary institutional attributes.**

| **Entity** | **Number of licenses** | **Percentage of total %** |
| --- | --- | --- |
| Research institutes | 78 | 18.14% |
| Universities | 94 | 21.86% |
| Enterprises | 258 | 60.00% |
