# Supplementary material for: Trends of laboratory nonhuman primate licensing in China between 2020 and 2024: A national database analysis
Source: PLoS One. 2026 May 12;21(5):e0348130. doi: 10.1371/journal.pone.0348130 (PMC13166922; doi:10.1371/journal.pone.0348130)
Supplement: S5 Table — (DOCX) [file pone.0348130.s005.docx]

**S5 Table. Principal laws and regulations relating to the management of laboratory primate in China.**

| **Name** | **Issued year** | **Issued by** | **Applicability** |
| --- | --- | --- | --- |
| Wildlife Protection Law of the PRC | 1988 | National People's Congress | Wildlife protection and management |
| Statute on the Administration of Laboratory Animals | 1988 | MOST | Administration of laboratory animals |
| Entry and Exit Animal and Plant Quarantine Law of the PRC | 1991 | National People's Congress | Cross-border animal/plant quarantine |
| Animal Epidemic Prevention Law of the PRC | 1997 | National People's Congress | Animal epidemic prevention and control |
| Regulation on the Management of Laboratory Animal Quality Control | 1997 | MOST | Quality control of laboratory animals |
| Regulation on the Management of Laboratory Animal Licensing System | 2001 | MOST | Administration of laboratory animal licenses |
| Guidelines on the Humane Treatment of Laboratory Animals | 2006 | MOST | Humane treatment of laboratory animals |
| Biosafety Law of the PRC | 2020 | National People's Congress | Biosafety risk prevention and control |
| Legal Norms for official veterinarians to perform their duties | 2020 | Ministry of Agriculture and Rural Affairs | Standardization of official veterinarians' duties |
| Ethical Guidelines for Human–Nonhuman Animal Chimera Research | 2021 | MOST and National Health Commission | Ethical norms for human-nonhuman animal chimera research |
| Measures for Science and Technology Ethics Reviews | 2023 | MOST | Ethical review of scientific and technological activities |
